# Biological, Functional and Genetic Characterization of Bone Marrow-Derived Mesenchymal Stromal Cells from Pediatric Patients Affected by Acute Lymphoblastic Leukemia

**DOI:** 10.1371/journal.pone.0076989

**Published:** 2013-11-07

**Authors:** Antonella Conforti, Simone Biagini, Francesca Del Bufalo, Pietro Sirleto, Adriano Angioni, Nadia Starc, Giuseppina Li Pira, Francesca Moretta, Alessandra Proia, Benedetta Contoli, Silvia Genovese, Claudia Ciardi, Maria Antonietta Avanzini, Vittorio Rosti, Francesco Lo-Coco, Franco Locatelli, Maria Ester Bernardo

**Affiliations:** 1 Department of Pediatric Hematology/Oncology, IRCCS Bambino Gesù Children's Hospital, Rome, Italy; 2 Cytogenetics and Molecular Genetics Unit, IRCCS Bambino Gesù Children's Hospital, Rome, Italy; 3 Laboratory of Neuro-Oncohematology, Fondazione Santa Lucia, University of Tor Vergata, Rome, Italy; 4 Department of Pediatric Hematology/Oncology, Fondazione IRCCS Policlinico San Matteo, Pavia, Italy; 5 Centro per lo Studio e la Cura della Mielofibrosi, Laboratori Sperimentali di Ricerca, Area di Biotecnologie, Fondazione IRCCS Policlinico San Matteo, Pavia, Italy; 6 University of Pavia, Pavia, Italy; Josep Carreras Leukaemia Research Institute, University of Barcelona, Spain

## Abstract

Alterations in hematopoietic microenvironment of acute lymphoblastic leukemia patients have been claimed to occur, but little is known about the components of marrow stroma in these patients. In this study, we characterized mesenchymal stromal cells (MSCs) isolated from bone marrow (BM) of 45 pediatric patients with acute lymphoblastic leukemia (ALL-MSCs) at diagnosis (day+0) and during chemotherapy treatment (days: +15; +33; +78), the time points being chosen according to the schedule of BM aspirates required by the AIEOP-BFM ALL 2009 treatment protocol. Morphology, proliferative capacity, immunophenotype, differentiation potential, immunomodulatory properties and ability to support long-term hematopoiesis of ALL-MSCs were analysed and compared with those from 41 healthy donors (HD-MSCs). ALL-MSCs were also genetically characterized through array-CGH, conventional karyotyping and FISH analysis. Moreover, we compared ALL-MSCs generated at day+0 with those isolated during chemotherapy. Morphology, immunophenotype, differentiation potential and *in vitro* life-span did not differ between ALL-MSCs and HD-MSCs. ALL-MSCs showed significantly lower proliferative capacity (p<0.001) and ability to support *in vitro* hematopoiesis (p = 0.04) as compared with HD-MSCs, while they had similar capacity to inhibit *in vitro* mitogen-induced T-cell proliferation (p = N.S.). ALL-MSCs showed neither the typical translocations carried by the leukemic clone (when present), nor other genetic abnormalities acquired during *ex vivo* culture. Our findings indicate that ALL-MSCs display reduced ability to proliferate and to support long-term hematopoiesis *in vitro*. ALL-MSCs isolated at diagnosis do not differ from those obtained during treatment.

## Introduction

Acute lymphoblastic leukemia (ALL) is the most common malignancy of childhood and arises from clonal proliferation of lymphoid precursors replacing normal hematopoietic cells in the bone marrow (BM) [1], [2]. Despite great advances in the treatment of the disease, which have led to current survival rates higher than 80%, pathogenic mechanisms of ALL still remain largely unknown [1], [2]. Although many recurrent genetic and chromosomal alterations, which are known to contribute to the origin of childhood leukemia, have been described in hematopoietic stem cells (HSCs) of ALL patients [2]–[4], a role played by the BM microenvironment, the essential framework where survival, proliferation and differentiation of HSCs take place, cannot be excluded and, indeed, it has been proposed by some authors [5]–[7].

A key component of the BM microenvironment is represented by mesenchymal stromal cells (MSCs); they are multipotent cells, with an extensive self-renewal capacity, that can differentiate into several mesenchymal lineages, such as bone, adipose tissue, cartilage, tendon and muscle [8]–[10]. MSCs give a substantial contribution to the creation of the HSC *niche*, through both cytokine secretion and cell-to-cell interaction, and play a crucial role in the development and differentiation of the hematopoietic system [11]–[12]. Moreover, MSCs exert peculiar immunomodulatory effects on all cells involved in the immune response, both *in vivo* and *in vitro*, through mechanisms which are not yet completely elucidated [13]–[15]. In clinical settings, MSCs proved to be able to facilitate the engraftment of HSCs after T-cell depleted, HLA-haploidentical HSC transplantation (HSCT) and to rescue patients with steroid-resistant, acute graft-versus-host disease (GvHD) [16]–[18]. Moreover, MSCs have been successfully employed in patients with chronic inflammatory diseases, such as Crohn's disease [19], [20]. In view of their properties and broad potential for clinical application, many efforts are being undertaken to understand the role of MSCs also in hematological malignancies [21]–[24].

In order to achieve more insights into this issue, we proceeded to phenotypically, functionally and genetically characterize BM-derived MSCs isolated from paediatric patients affected by ALL (ALL-MSCs). We compared these cells with MSCs isolated from BM of healthy donors (HD-MSCs); we also evaluated whether repeated administrations of chemotherapy in these patients might affect MSC biological properties. For this latter purpose, we isolated and characterized ALL-MSCs during patient treatment at different time-points and compared results with those obtained at the onset of the disease.

## Patients and Methods

### ALL patients and healthy donors

Forty-five children (22 males, 23 females; median age 5 years, range 1–17), diagnosed with ALL at the Department of Pediatric Hematology-Oncology of the IRCCS Bambino Gesù Children's Hospital between August 2010 and June 2012 and treated according to the Italian Association of Pediatric Hematology and Oncology (AIEOP) - Berlin/Frankfurt/Münster (BFM) ALL 2009 protocol, were included in the study. The diagnosis was based on BM-aspirate morphology, cell immunophenotype determined by means of flow cytometry, and cytogenetic analysis [25], [26]. Three patients were affected by T-cell precursor ALL, whereas the remaining 42 children had B-cell precursor ALL. Table 1 details patient characteristics at diagnosis. MSCs were isolated and expanded *ex vivo* from BM both at diagnosis, *i.e.* day+0 (38 patients), and at subsequent time points of treatment, *i.e.* days +15 (25 children), +33 (31 patients) and +78 (27 patients). The study was approved by the Institutional Ethical Committee and written informed assent/consent was obtained from patients and/or their legal guardians.

**Table 1:** Characteristics of Acute Lymphoblastic Leukemia (ALL) patients at time of diagnosis.

**Total N. of ALL patients**	45
**Median age (range)**	5 years (1–17)
**Gender**	
**Males**	22
**Females**	23
**ALL immunophenotype**	
Pro-B	3
Pre-B common	38
Mature B	1
T	3
**Genetic aberrations**	
None	31
t(4;11) – MLL/AF4	2
t(12;21) – TEL/AML1	9
t(1;19) – E2A/PBX1	1
t(8;14)	1
SIL/TAL	1
**Disease localization at diagnosis**	
BM	45
CNS	1
Mediastinum	1

BM: bone marrow; CNS: central nervous system.

As controls, after obtaining written informed consent, we used MSCs isolated from residual cells of 41 healthy donors (18 males, 23 females, median age 21 years, range 5–34), who donated BM for transplantation at the Bambino Gesù Children's Hospital.

### Isolation and culture of BM-derived ALL- and HD-MSCs

Mononuclear cells were isolated from BM aspirates (10 ml) of ALL patients and HDs by density gradient centrifugation (Ficoll 1.077 g/ml; Lympholyte, Cedarlane Laboratories Ltd., The Netherlands) and plated in non-coated 75–175 cm^2^ tissue culture flasks (BD Falcon, NJ, USA) at a density of 160,000/cm^2^ in complete culture medium: DMEM (Euroclone, Milan, Italy) supplemented with 10% fetal bovine serum (FBS; Gibco, Life Technologies Ltd, Paisley, UK), penicillin 50 U/ml, 50 mg/ml streptomycin and 2 mM L-glutamine (Euroclone). Cultures were maintained at 37°C in a humidified atmosphere, containing 5% CO_2_. After 48-hour adhesion, non-adherent cells were removed and culture proceeded with culture medium being replaced twice a week. MSCs were harvested, after reaching ≥80% confluence, using Trypsin (Euroclone), and were propagated at 4,000 cells/cm^2^.

### Characterization of *ex-vivo* expanded ALL- and HD-MSCs

#### Proliferative capacity

Cell growth was analyzed by direct cell counts and population doublings (PDs) were determined at each passage. The number of PDs was calculated for each MSC sample by using the formula log_10_(N)/log_10_(2) where N means cells harvested/cells seeded; results were expressed as PD from passage (P) 1 to P5 [27].

#### Immune-phenotype

ALL- and HD-MSCs were phenotypically characterized by flow cytometry at all time points. Fluorescein isothiocyanate (FITC)- or phycoerythrin (PE)-conjugated monoclonal antibodies specific for CD13, CD14, CD34, CD45, CD73, CD80, CD90, class I-HLA and HLA-DR, CD73, **CD105** (BD PharMingen, San Diego, CA) were used. Appropriate, isotype-matched, non-reactive fluorochrome-conjugated antibodies were employed as controls. Analysis of cell populations was performed by means of direct immunofluorescence with a FACSCanto flow-cytometer (BD PharMingen) and data were calculated using the FACSDiva software (Tree Star, Inc. Ashland, OR).

#### Differentiation capacity

The osteogenic differentiation capacity of ALL-MSCs isolated at any time point was assessed at P2 and at P6–8 by incubating cells with αMEM (Euroclone), 10% FBS, penicillin 50 U/ml, 50 mg/ml streptomycin, and 2 mM L-glutamine supplemented with 10^−7^M dexamethasone, 50 mg/ml L-ascorbic acid and, starting from day +7 of the culture, 5 mM ß-glycerol phosphate (Sigma-Aldrich, St Louis, MO) was added to the medium. Adipogenic differentiation was evaluated at P2 and at P6–8 by incubating cells with αMEM, 10% FBS, penicillin 50 U/ml, 50 mg/ml streptomycin, and 2 mM L-glutamine supplemented with 10^−7^M dexamethasone, 50 mg/ml L-ascorbic acid, 100 mg/ml insulin, 50 mM isobutyl methylxanthine, 0,5 mM indomethacin (Sigma-Aldrich) and 5 mM b-glycerol phosphate. Both osteogenic and adipogenic cultures were incubated for at least two weeks before evaluating differentiation. To detect osteogenic differentiation, cells were stained for alkaline phosphatase (AP) activity using Fast Blue (Sigma-Aldrich) and for calcium deposition with Alizarin Red (Sigma-Aldrich). Adipogenic differentiation was evaluated through the morphological appearance of fat droplets stained with Oil Red O (Sigma-Aldrich).

To quantify AP expression, after washing with PBS, 400 µl 0.05N NaOH in ethanol were added to each well and AP extraction was measured spectrophotometrically at 550 nm. DNA was extracted with QIAmp DNA Mini kit (QIAGEN, Netherlands), following manufacturer's instructions. For adipogenic quantification, after staining with Oil Red O, 300 µl of ethanol (100%) were added to each well to extract the Oil Red O from the cells. Then, the amount of Oil Red O released was determined spectrophotometrically at 550 nm with a reference of 650 nm and compared to an Oil Red O standard titration curve. The amount of released Oil Red O or AP was divided by the amount of DNA collected from the same wells [28].

### Senescence assay and cell cycle analysis

ALL-MSCs and HD-MSCs were maintained in culture until reaching senescence. The life-span *in vitro* of HD-MSCs and ALL-MSCs was defined by the number of passages in culture before observation of senescence. MSCs were closely monitored during senescence for up to 8–12 weeks before interrupting the cultures, in order to reveal any change in morphology and/or proliferation rate [29]. Senescence of MSCs was also assessed by staining with a senescence β-galactosidase (SA-β-gal) Staining Kit (Cell Signaling Technology, Danvers, MA), according to manufacturer's instructions, and evaluated by direct-light microscopy.

For cell cycle analysis, MSCs were harvested by trypsinization, washed in cold PBS, fixed in 50% PBS and 50% acetone/methanol for at least 1 h and, after removing alcoholic fixative, stained with a solution containing 50 µg/ml Propidium Iodide (BD PharMingen) and 100 µg/ml RNase (Sigma-Aldrich) for 30 min at room temperature in the dark. Samples were then evaluated by flow-cytometry (FACSCanto flow-cytometer; BD PharMingen).

### *In vitro* PBMC proliferation assay with phytohemagglutinin

Peripheral blood mononuclear cells (PBMCs) were obtained by conventional Ficoll separation from heparinized peripheral blood samples from 10 healthy donors, who gave informed consent for this study; cells were employed on the same day of collection.

The proliferation of PBMCs from healthy donors in RPMI 1640 medium (Gibco, Life Technologies Ltd) supplemented with 10% FBS, in response to phytohemagglutinin (PHA-P; Sigma-Aldrich), either in the presence or absence of BM-derived MSCs, was performed in triplicate in flat-bottom 96-well tissue culture plates (BD Falcon, Franklin Lakes, NJ, USA). Briefly, ALL-MSCs and HD-MSCs were seeded at MSC∶PBMC ratios of 1∶2 and 1∶10 per well and allowed to adhere overnight before adding 10^5^ PBMCs per well with or without PHA (4 µg/ml). After 3-day incubation at 37°C in a humidified 5% CO_2_ atmosphere, cultures were pulsed with ^3^H-thymidine (1 µg Ci/well, specific activity 6.7 Ci/mmole, Perkin Elmer, Waltham, MA) and harvested after 18 hours. ^3^H-thymidine incorporation was measured by standard procedure with Microbeta Trilux 1450 instrument (Perkin Elmer); results were expressed as percentage of proliferation. All the experiments were performed in an allogeneic setting (i.e. HD-PBMCs/ALL-MSCs and HD-PBMCs/HD-MSCs).

### Measurement of growth factors and cytokines by ELISA

The concentration of IL2, IL6, IL10, TGFβ, GM-CSF and IFNγ in supernatants of co-cultures of both HD-MSCs and ALL-MSCs (isolated at day+0) with PBMCs, after 72-hour incubation with PHA, was quantified by means of commercially available ELISA kits obtained from Mabtech (Nacka strand, Sweden), whereas the analysis of HGF, Galectin-1 and PGE2 content was performed using ELISA kits obtained from R&D System (Minneapolis, MN, USA), following the manufacturer's instructions. Plates were then read either at 405 nm (for ALP-conjugated antibodies) or at 450 nm (for HRP-conjugated antibodies) through Envision Multilabel Reader (Perkin Elmer).

### MSC-mediated support of long-term hematopoiesis

The capacity of ALL-MSCs (isolated at diagnosis) and HD-MSCs to support normal hematopoiesis *in vitro*, was assessed as previously described [30]. Briefly, confluent MSCs were trypsinized, irradiated (30 Gy) by the use of a Cesium irradiator, and subcultured at a concentration of 3×10^4^ MSCs/well in 96-well plates. After MSC overnight adhesion, plates were recharged with 1000 CD34+ cells/well derived from third-party HDs in LTC-IC Myelocult medium (Stemcell Technologies) with 10^−6^ M hydrocortisone and incubated for 5 weeks with weekly culture refeeding. Experiments were performed in triplicate. At the end of culture, CD34+ cells were collected and plated into 30 mm petri dishes with 1 mL/well complete methylcellulose medium (Methocult GF H4534, StemCell Technologies) and maintained at 37°C in 5% CO_2_ for 14 days. Results were expressed as mean and range of total yield of colony-forming-units (CFUs). Experiments were performed with MSCs obtained from 7 ALL patients (at d+0) and 6 HDs at passages between 2 and 4.

### Genetic characterization of ALL-MSCs

#### Molecular karyotyping

Molecular karyotyping was performed on ALL-MSCs through array comparative genomic hybridization (array-CGH) with the Agilent kit (Human Genome CGH Microarray, Agilent Technologies, Santa Clara, CA). The array-CGH platform is a 60-mer oligonucleotide-based microarray that allows a genome-wide survey and molecular profiling of genomic aberrations with a resolution of about 41 kb (kit 60K). DNA was extracted from MSCs of the patients with QIAamp® DNA Blood Kit (QIAGEN) according to the manufacturer's instructions. DNA (1 µg) from MSCs and controls of the same sex (control DNA, Promega, Madison, WI, USA) were processed according to the protocol (Agilent Oligonucleotide Array-Based CGH for Genomic DNA Analysis – Version 6.2.1, February 2010). The array was analyzed through the Agilent Scanner and the Feature Extraction software (v10.7.3.1) and Agilent Genomic Workbench Lite Edition 6.5.0.18.

#### Cytogenetic analysis by conventional karyotype

ALL-MSCs were also analyzed by conventional karyotyping and FISH analysis. Prior to harvest, ALL-MSCs at P3–P7 were incubated at 37°C with colcemid (IrvineScientific, Santa Ana, CA) at 1 µg/ml final concentration for 5 hours. The cells were fixed and spread according to standard procedures. Metaphases of cells were GTG-banded and karyotyped in accordance with the International System for Human Cytogenetic Nomenclature recommendations (ISCN, 2009).

#### Fluorescent in situ Hybridization (FISH) for TEL/AML and MLL/AF4

Fluorescent *in situ* hybridization (FISH) with TEL/AML [t(12;21)] probes (POSEIDON™ DNA PROBES, KREATECH, Amsterdam, NL) was performed on fixed metaphase chromosomes and interphase cells obtained from ALL-MSC samples isolated from 3 patients found to carry the t(12;21) translocation, according to manufacturer's instructions. MLL rearrangements were analyzed on ALL-MSC samples isolated from the 2 patients carrying the t(4;11) translocation using the LSI MLL Dual Color Break Apart Rearrangement Probe. At least 500 nuclei were analyzed. The slides were analyzed in a fluorescence microscope equipped with appropriate filters using the ISIS-software (Metasystems).

#### RQ-PCR for MLL/AF4

The ALL-MSC samples isolated from the 2 patients carrying the t(4;11) translocation in their leukemic clone were also analyzed by RQ-PCR for MLL/AF4 detection. Total RNA extraction was performed by the guanidine thiocyanate procedure [31], and RQ-PCR was done as previously described [32]. Primer sequences were as follows: 5′-CCCAAGTATCCCTGTAAAACAAAAA-3′ and 5′-GATGGAGTCCACAGGATCAGAGT-3′(forward), 5′-GAAAGGAAACTTGGATGGCTCA-3′ (reverse), and 5′CATGGCCGCCTCCTTTGACAGC-3′ (probe).

### Statistical analysis

Comparisons on the equality of means of population doublings and CFU were performed by using the Student's t-test, assuming paired data. The two-sample test of proportion was used to compare data expressed in percentages (PHA-induced PBMC proliferation). For both tests, a p-value lower than 0.05 was considered to be significant. Statistical analysis was performed using the Stata/IC 11.0 software package. Data were analyzed on December, 2012.

## Results

### Characterization of BM-derived ALL-MSCs

MSCs were successfully isolated and propagated *in vitro* from the BM of all patients, both at diagnosis and at the subsequent time-points of treatment, and were compared with MSCs obtained from the BM of 41 HDs. In particular, as far as ALL patients are concerned, MSCs were isolated from 38 patients at diagnosis (d+0), 25 children at day +15, 31 at day +33 and 27 at day +78, the time points being chosen according to the schedule of BM aspirates required by the AIEOP-BFM ALL 2009 treatment protocol. Both ALL-MSCs and HD-MSCs were then characterized by morphology, proliferative capacity, differentiation potential and immunophenotype.

**Figure 1:**
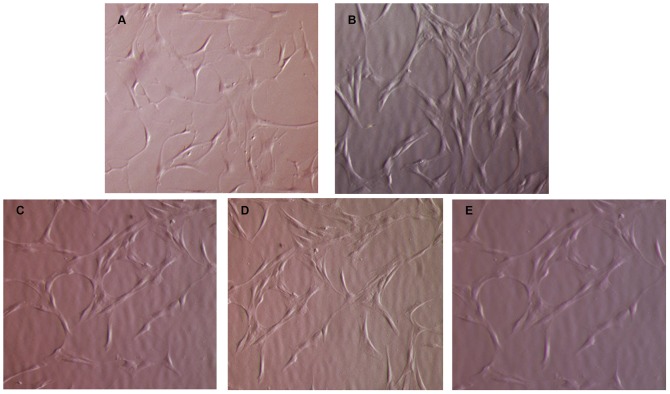
Morphology of culture-expanded HD-MSCs and ALL-MSCs. MSCs from both patients and donors display the characteristic spindle-shaped morphology. **A:** morphology of HD-MSCs from donor N.3; **B:** morphology of ALL-MSCs from patient N.7 at diagnosis (d+0); **C,D,E:** morphology of ALL-MSCs from patient N.7 at subsequent time-points after beginning of treatment, *i.e.* d+15, +33 and +78, respectively. Magnification ×10.

**Figure 2:**
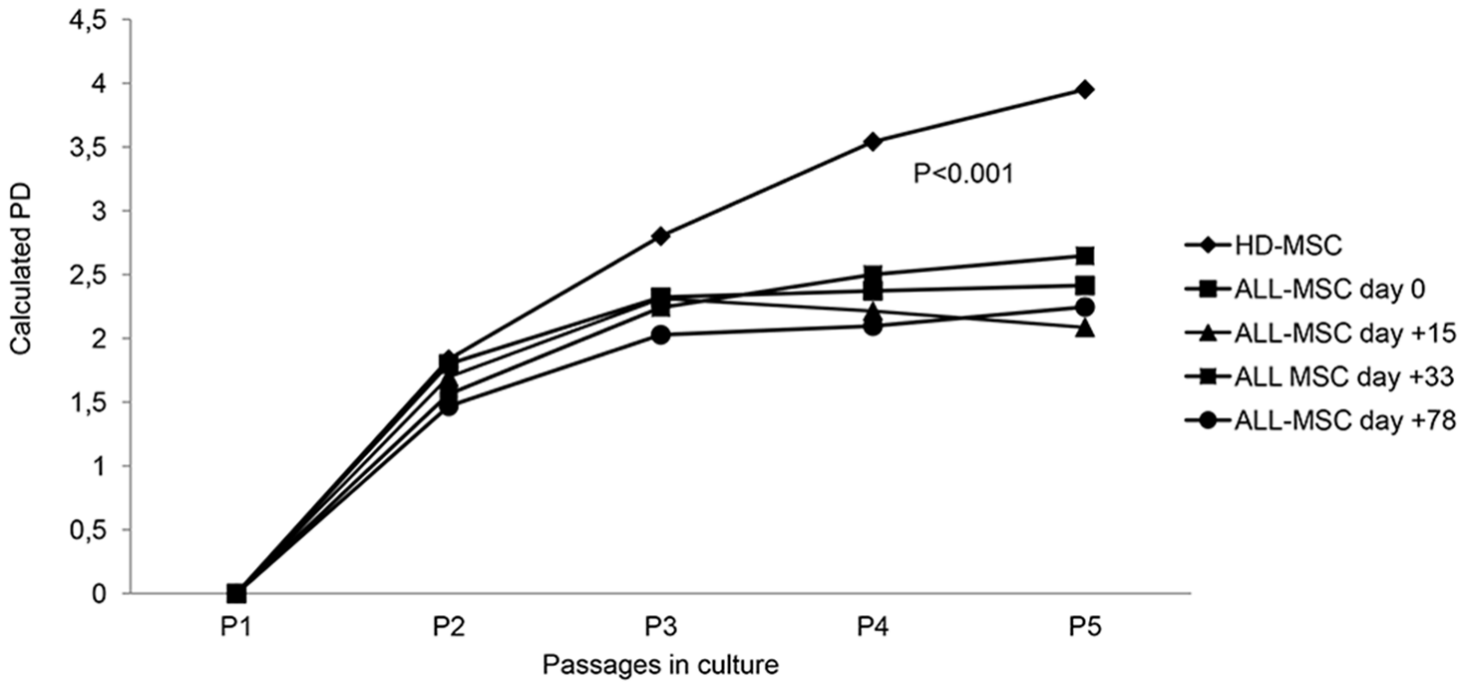
Proliferative capacity of ALL-MSCs as compared with HD-MSCs. Calculated cumulative population doublings (PDs) from P1 to P5 of MSCs isolated from HDs and from ALL patients at diagnosis and at following time-points of treatment, namely day+15, +33 and +78. The data represent the mean of 10 HD-MSCs and 10 ALL-MSCs. P values less than 0.05 were considered to be statistically significant.

MSCs isolated from ALL patients at any time point considered displayed the characteristic spindle-shaped morphology, similar to that shown by HD-MSCs (Fig. 1). In order to compare their proliferative capacity, ALL-MSCs and HD-MSCs were plated in parallel utilizing the same culture conditions; the number of PDs through at least five culture passages was calculated. Irrespective of the time point at which they were generated, ALL-MSCs showed a significantly lower proliferative capacity, as compared with HD-MSCs. In particular, the calculated cumulative PDs from P1 to P5 were as follows: HD-MSCs = 12.12 (SD ±1.09); ALL-MSCs at d+0 = 8.89 (SD ±0.87; P<0.001); ALL-MSCs at d+15 = 8.29 (SD ±0.91; P<0.001); ALL-MSCs at d+33 = 8.94 (SD ±1.07; P<0.001); ALL-MSCs at d+78 = 7.81 (SD ±0.56; P<0.001, Fig. 2).

**Figure 3:**
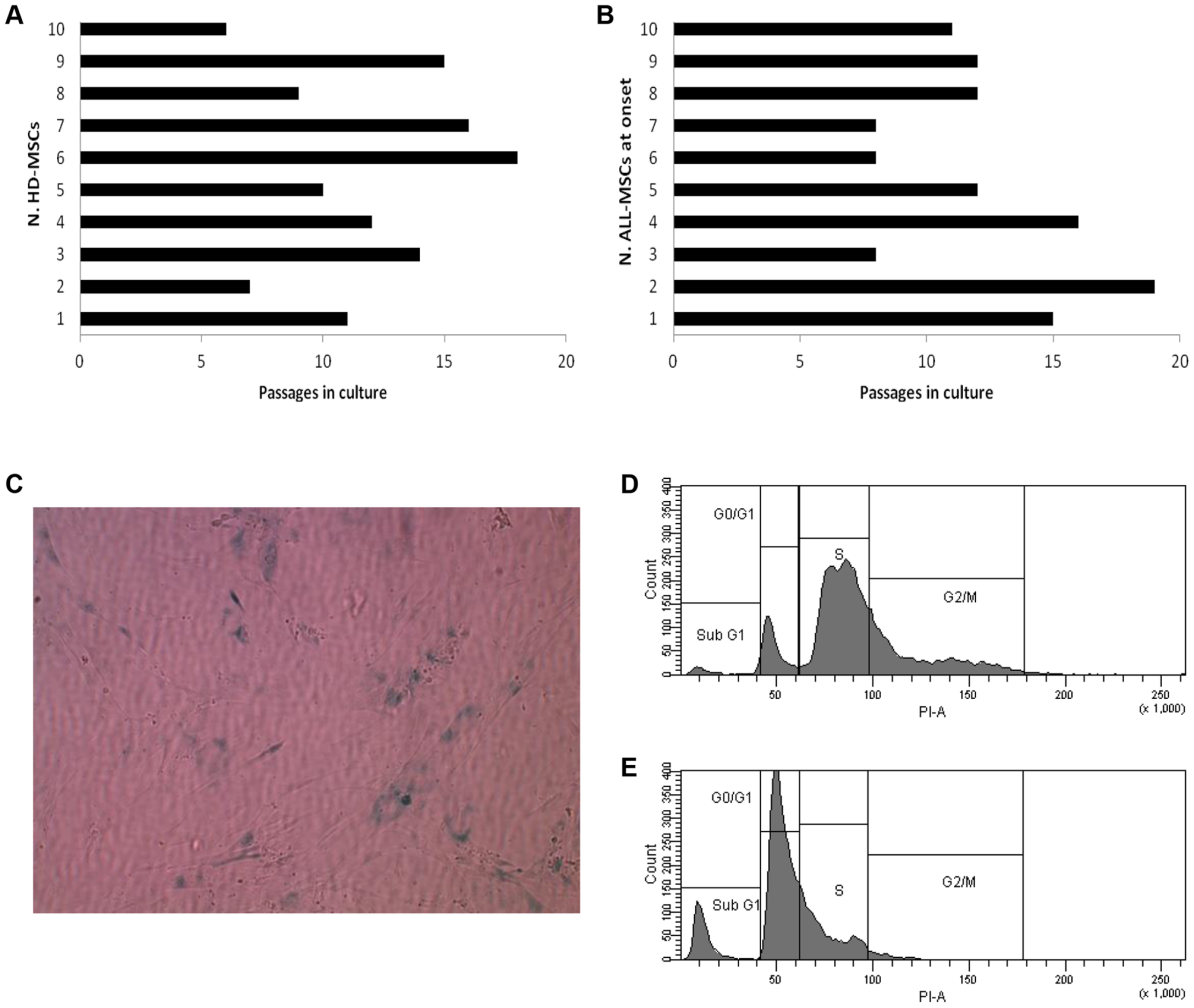
*In vitro* life-span and senescence of ALL-MSCs as compared with HD-MSCs. *In vitro* life-span of HD-MSCs (**A**) and ALL-MSCs at diagnosis (**B**), defined as number of passages before observation of senescence, from 10 representative donors and patients (#1–10). A large variability among HDs and patients was observed. (**C**): ß-Galactosidase staining of ALL-MSCs from patient #2 at passage (P) 18, isolated at day+0. Senescence of the cells is indicated by the positivity for the staining. **D and E:** cell cycle analysis performed by flow-cytometry on ALL-MSCs from patient #2 at early (P2) and late (P18) passages in culture. At P2 (D), the percentage of proliferating cells (in S phase) is 56.6%, whereas cells in G0/G1, G2 phase are 11.1 and 29.2%, respectively. The percentage of apoptotic cells in subG1 phase is 2%. At P18 (E), when cells become senescent, the percentage of proliferating cells decreases to 26.5%, whereas cells in G0/G1, G2 and subG1 phase are 57.5%, 2.6% and 12.3%, respectively.

With respect to the length of time during which MSCs proliferated *in vitro* (life-span), as shown in Figure 3A–B, HD-MSCs ceased to grow between P7 and P18, whereas ALL-MSCs isolated at diagnosis could be expanded *ex vivo* until P8–P19. This indicates a similar *in vitro* life-span of the cultures, despite the expected wide variability among different subjects, in analogy with previously reported data [29]. Superimposable results were obtained with ALL-MSCs isolated at subsequent time points (data not shown). Both HD- and ALL-MSCs were observed in culture during their senescence phase for up to 8–12 weeks and they did not show any sign of transformation or cell growth acceleration. Senescence of the cultures was indicated by the decrease in MSC proliferative capacity, finally leading to cell-cycle arrest, as well as by the positivity for β-Gal staining (Fig. 3C). Moreover, MSCs acquired a typical flat, round shape in culture which was evaluated by phase contrast microscopy. Cell cycle analysis performed by flow-cytometry demonstrated that while at P2 (Fig. 3D) the percentage of proliferating MSCs (in S phase) is 56.6%, it lowers to 26.5% at P18 (Fig. 3E), when cells become senescent. Accordingly, the percentage of apoptotic cells in subG1 phase is 2% at P2, whereas it becomes 12.3% at P18.

**Figure 4:**
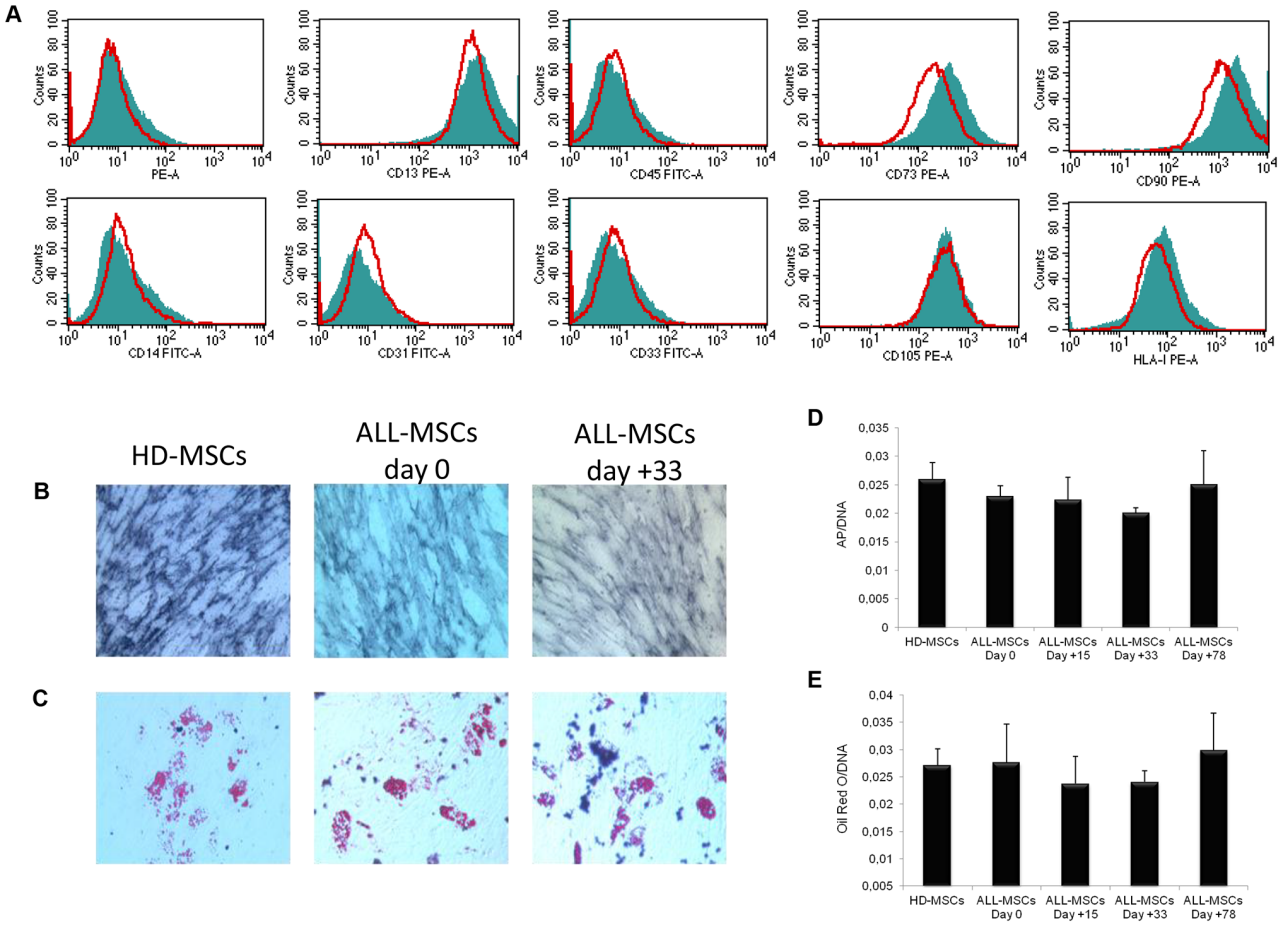
A: Phenotypic and differentiation characterization of ALL-MSCs. Immunophenotype of culture-expanded HD-MSCs and ALL-MSCs at diagnosis from a representative sample. In red HD-MSCs and in green ALL-MSCs. Histograms of surface marker expression of HD-MSCs and ALL-MSCs are similar: both types of MSCs are positive for CD13, CD73, CD90, **CD105** and HLA-I surface antigens and negative for CD14, CD31, CD33 and CD45 molecules. Immunophenotype of ALL-MSCs at subsequent time-points of the disease and treatment was superimposable. **B and C: Osteogenic and adipogenic differentiation capacity of MSCs isolated from one representative HD and one ALL patient at diagnosis (d+0) and at d+33.** In B, the differentiation into osteoblasts is demonstrated by the histological detection of alkaline phosphatase (AP) activity (purple reaction); magnification ×20. In C, the differentiation into adipocytes is revealed by the formation of lipid droplets stained with Oil Red O; magnification ×20. **D and E: Quantification of the osteogenic and adipogenic differentiation capacity of MSCs isolated from one representative HD and one ALL patient at diagnosis (d+0) and subsequent time-points (d+15, +33 and +78).** In D the amount of AP is related to the amount of DNA extracted from the same wells and is expressed in mOD/min/mg; in E the amount of Oil Red O is related to the DNA content of the same wells and is expressed as mg/mg. Each bar represents the mean +/−SD of the results from three different experiments, each performed in triplicate.

ALL-MSCs from all samples at any time-point were immunophenotypically characterized by flow-cytometry at P2 or P3. Their phenotype was in agreement with previous publications [18], [19], [32] and comparable with that of HD-MSCs. In particular, in ALL-MSCs both at diagnosis and at later time points, contamination with hematopoietic cells was no longer detectable by P3 (negativity for CD14, CD33, CD45, CD80 and HLA-DR) and more than 98% of cells expressed the surface marker pattern (positivity for CD13, CD73, CD90, CD105 and HLA-I) typical of MSCs (Fig. 4A).

To examine the differentiation capacity of MSCs isolated from ALL patients, as compared with HD-MSCs, cells were induced into osteoblasts and adipocytes and examined by histological staining. As shown in Figure 4B–C, both HD-MSCs and ALL-MSCs at any time-point were able to differentiate into osteoblasts, as demonstrated by the histologic detection of alkaline phosphatase activity (Fig. 4B) and calcium deposition (data not shown), as well as into adipocytes, as revealed by the formation of lipid droplets (Fig. 4C). The differentiation ability of ALL-MSCs into osteoblasts and adipocytes was also quantified spectrophotometrically, as shown in Figure 4 D and E, respectively. Moreover, the differentiation capacity was maintained, unmodified, until P8 (data not shown).

No differences were found in the characterization of MSCs derived from B- and T-cell precursor ALL (data not shown).

### Effect of ALL-MSCs on PHA-induced PBMC proliferation and cytokine analysis

**Figure 5:**
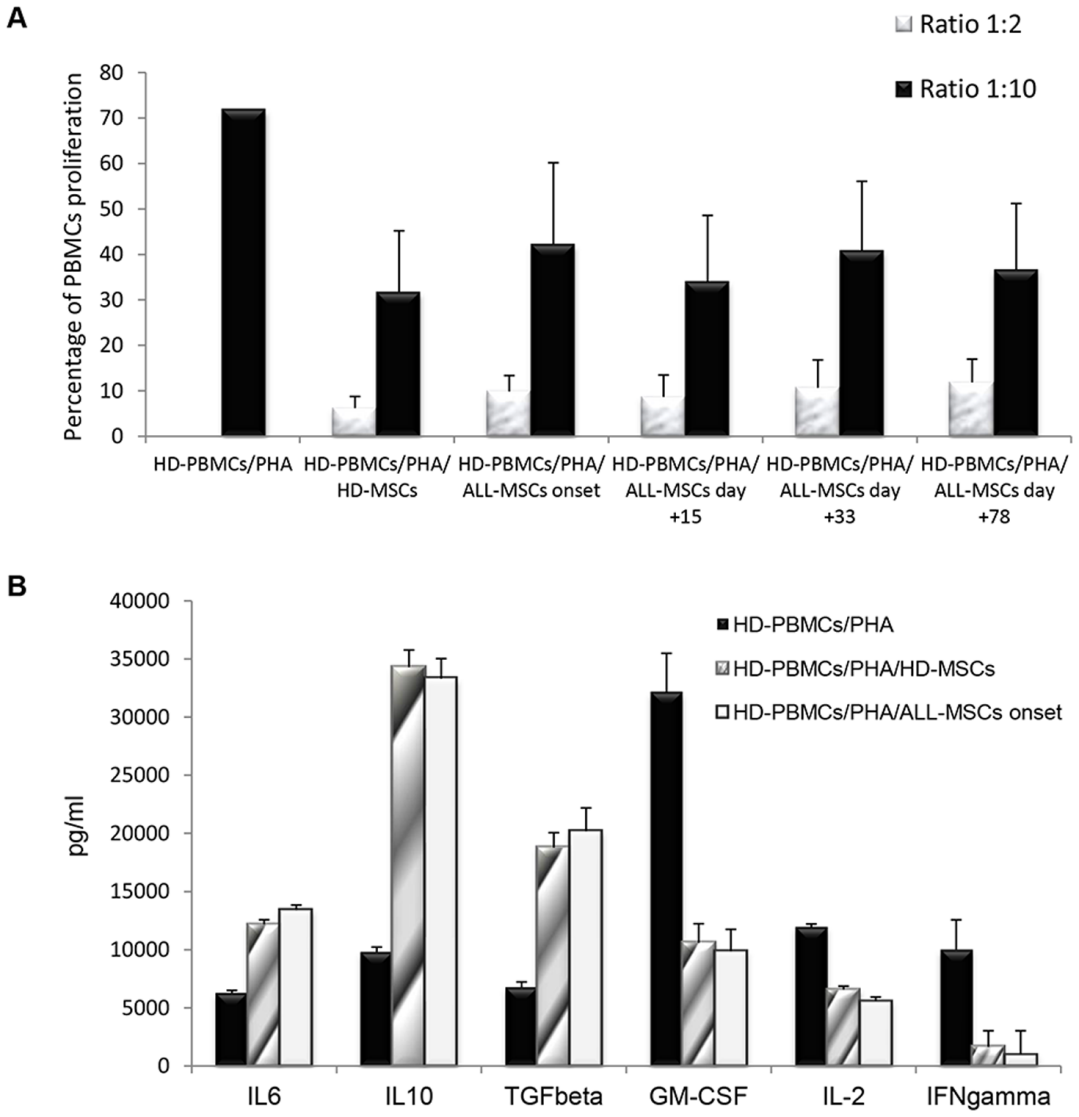
A: *In vitro* immunomodulatory effect of HD-MSCs and ALL-MSCs on HD-PBMCs. The graph shows the proliferation of healthy donor peripheral blood mononuclear cells (HD-PBMCs) stimulated with phytohemagglutinin (PHA) either in the presence or in the absence of HD-MSCs or ALL-MSCs, generated at disease onset and subsequent time-points of treatment (day+15; day+33; day+78). Each bar represents the percentage of proliferation of 10^5^ PBMCs, in the presence of two different MSC∶PBMC ratios (MSC∶PBMC ratio of 1∶2 and 1∶10), calculated by measuring ^3^H-thymidine incorporation after 3-day co-culture. The counts per minute (cpm) values at each cell concentration were normalized to the cpm of PBMCs without MSCs in each experiment. Each bar represents the mean ± SD of multiple experiments (each point being in triplicate) with MSCs obtained from 10 ALL patients (at each time-point) and 10 HDs. **B:** **Quantification of cytokines and growth factors in supernatants of co-cultures of HD-MSCs and ALL-MSCs (isolated at day+0) with PBMCs, after 72-hour incubation with PHA.** An increase in anti-inflammatory cytokines (*i.e.* IL6, IL10 and TGFb) was detected in the presence of both HD-MSCs and ALL-MSCs, as compared with PHA-stimulated PBMC cultures. A decrease in pro-inflammatory cytokines and growth factors (GM-CSF, IL2 and IFNg) was revealed in supernatants collected from the same co-cultures. Each bar represents the mean +/−SD of the results from experiments performed with the same HD- and ALL-MSC samples employed in the PBMC proliferation assays. Results are expressed as pg/ml.

In order to evaluate the immunomodulatory capacity of *ex vivo* expanded MSCs, isolated both from HDs and from ALL patients, we measured PBMC proliferation induced by PHA either in the presence or in the absence of MSCs in an allogeneic setting. In agreement with previously reported studies [13], [14], HD-MSCs proved to exert a strong *in vitro* inhibitory effect on PHA-induced PBMC proliferation, with a mean percentage of proliferation in the presence of HD-MSCs of 6.2% (SD ±2.55) and 31.7% (SD ±13.5) at MSC∶PBMC ratios of 1∶2 and 1∶10, respectively; PBMC proliferation in the absence of MSCs was 71.8%. With regard to MSCs derived from patients, ALL-MSCs isolated at diagnosis were able to prevent proliferation of allogeneic PBMCs at a similar degree as compared with HD-MSCs with a mean percentage of proliferation of 9.8% (SD ± 3.50; P = 0.38) at MSC∶PBMC ratio of 1∶2 and 42.2% (SD ±17.9; P = 0.31) at MSC∶PBMC ratio of 1∶10 (Fig. **5A**). Comparable results were obtained with ALL-MSCs isolated at any subsequent time-point considered (Table 2). In both patients and donors, a clear dose-dependent effect was observed in the ability of MSCs to inhibit mitogen-stimulated PBMC proliferation. These results indicate that MSCs isolated from the BM of ALL patients, both at diagnosis and at subsequent time-points, maintain their anti-proliferative effect on PHA-induced lymphocyte proliferation *in vitro* and that the chemotherapy administered during disease treatment does not affect this peculiar property of MSCs.

**Table 2:** Mean percentage of proliferation of healthy donor peripheral blood mononuclear cells (PBMCs) stimulated with PHA in the presence of HD-MSCs, as compared with ALL-MSCs generated at day+0; +15; +33; +78 at two different MSC∶PBMC ratios (1∶2; 1∶10).

	PBMC proliferation	P value	PBMC proliferation	P value
	*Ratio 1∶2*		*Ratio 1∶10*	
**HD-MSCs**	6.19 (SD ±2.55)	-	31.71 (SD ±13.49)	-
**ALL-MSCs d+0**	9.85 (SD ±3.50)	0.38	42.24 (SD ±17.93)	0.31
**ALL-MSCs d+15**	8.64 (SD ±4.82)	0.42	34.07 (SD ±14.50)	0.46
**ALL-MSCs d+33**	10.66 (SD ±6.12)	0.36	40.85 (SD ±15.23)	0.34
**ALL-MSCs d+78**	11.84 (SD ±5.12)	0.33	36.61 (SD ±14.58)	0.41

Each value represents the mean ± SD of multiple experiments (each point being in triplicate). PBMC proliferation stimulated with PHA in the absence of MSCs was 71.80%. P values less than 0.05 were considered to be statistically significant.

Furthermore, in order to better define the anti-inflammatory properties of HD-MSCs vs. ALL-MSCs and the mechanisms underlying MSC-mediated immuneregulatory effect, we measured cytokine and growth factor content in co-cultures of PHA-stimulated PBMCs with HD-MSCs and ALL-MSCs. As shown in Figure 5B, an increase in anti-inflammatory cytokines (*i.e.* IL6, IL10 and TGFβ) was detected in the presence of both HD-MSCs and ALL-MSCs, as compared with PHA-stimulated PBMC cultures; on the contrary, a decrease in pro-inflammatory cytokines and growth factors (GM-CSF, IL2 and IFNγ) was found in supernatants collected from co-cultures of PHA-stimulated PBMCs with HD-MSCs and ALL-MSCs. Moreover, we measured the concentration of other soluble factors known to be associated with the immuneregulatory/anti-inflammatory effect of MSCs, namely HGF, PGE2 and Galectin-1 and, as expected, these factors markedly increased in the presence of both HD- and ALL-MSCs (data not shown).

### Ability of ALL-MSCs to support long-term hematopoiesis

The capacity of MSCs to support hematopoiesis was evaluated in 7 ALL patients (at d+0) and 6 HDs at early passages. ALL-MSCs were significantly less capable to support long-term hematopoiesis *in vitro* as compared with HD-MSCs: mean of the total yield of colonies in case of ALL-MSCs 2.6 (range 0–5) and for HD-MSCs 4.7 (2–8) (p = 0.04). Most of the colonies were represented by CFU-GM; BFU-E were poorly grown both using HD-MSCs (median 0, range 0–1) and ALL-MSCs (median 0, range 0–1); CFU-GEMM were never observed. Cultures with ALL-MSCs from patients carrying cytogenetic abnormalities (n = 2 out of 7 examined) showed yield and frequency of CFU similar to those of patients without abnormalities, although the small number of experiments does not allow any meaningful statistical evaluation.

### Genetic profile of ALL-MSCs

In order to evaluate both ALL-MSC resistance to spontaneous transformation into malignant cells, a potential risk related to expansion procedures, and the possible presence of genetic alterations typical of leukemia cells, ALL-MSCs were genetically characterized through array-CGH, conventional karyotyping and FISH analysis.

**Figure 6:**
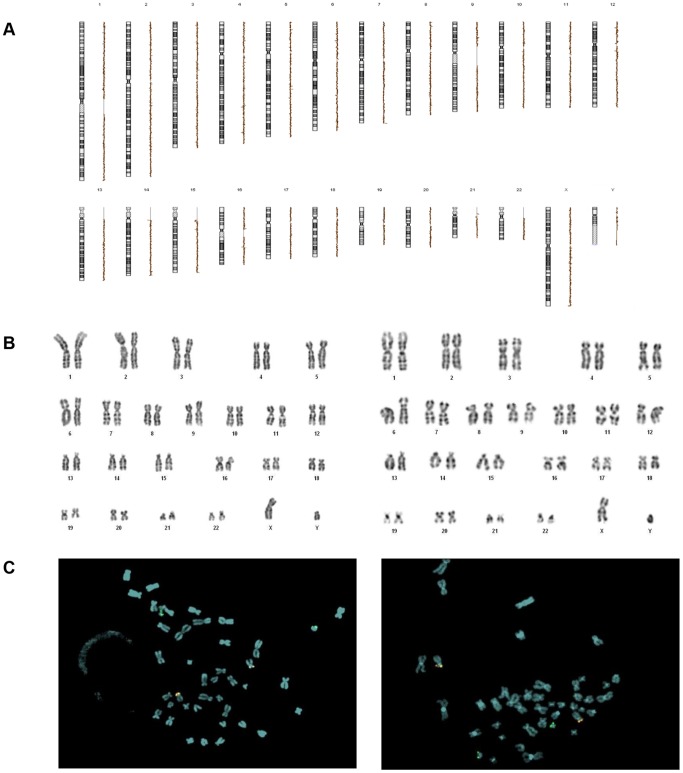
Genetic characterization of ALL-MSCs. **A:** array-CGH profiles of ALL-MSCs at P4 isolated at diagnosis (d+0) and at d+78; the profiles are linear and do not show unbalanced chromosomal rearrangements. **B:** Cytogenetic analysis through conventional karyotype of ALL-MSCs at P5 isolated at d+0 and d+78; the figure demonstrates the absence of chromosomal aberrations. **C:** FISH analysis with *TEL/AML* [t(12;21)] probes of ALL-MSCs from one of the patients carrying this specific translocation at P5; MSCs isolated at d+0 and d+78 are represented. The [t(12;21)] translocation was not found in *ex vivo* expanded MSCs. **D:** FISH analysis for MLL rearrangement of ALL-MSCs from one of the 2 patients carrying this specific translocation at P3; MSCs isolated at d+0 and d+78 are represented; the rearrangement was not found in *ex vivo* expanded MSCs.

Array-CGH profiles of *ex vivo* expanded ALL-MSCs did not show the presence of imbalanced chromosomal rearrangements; indeed, we could not detect any deletion or duplication of material both in ALL-MSCs isolated at diagnosis and in ALL-MSCs isolated at any subsequent time-point (Fig. 6A). These results indicate that ALL-MSCs do not show a propensity to undergo malignant transformation when forced in our *ex vivo* culture system, even after being exposed to repeated doses of vincristine, daunorubicine, cytosine-arabinoside, cyclophosphamide, 6-mercaptopurine and methotrexate, drugs which are known to induce DNA synthesis block and damage [34], [35].

Since array-CGH cannot unravel balanced chromosomal rearrangements, with the aim of broadening our genetic study on ALL-MSCs, we also performed conventional karyotyping and FISH analysis on the same ALL-MSC samples investigated by array-CGH. Cytogenetic analysis through conventional karyotyping did not show any chromosomal aberration in ALL-MSCs obtained both at diagnosis and at subsequent time-points of leukemia treatment (Fig. 6B). Moreover, the FISH analysis with *TEL/AML* [t(12;21)] probes performed on ALL-MSC samples isolated from 3 patients found carrying t(12;21) in the leukemic clone at diagnosis did not show the translocation at any time-point considered (Fig. 6C). Likewise, when *MLL* rearrangements were analyzed on ALL-MSC samples isolated from the 2 patients carrying the t(4;11) translocation, they were not found (Fig. 6D). The absence of the *MLL/AF4* fusion gene was also confirmed by real time PCR in both patients (data not shown).

## Discussion

In the present study, we isolated and propagated in culture MSCs derived from the BM of children affected by ALL with the aim of characterizing their *in vitro* biological and functional properties. For this purpose, ALL-MSCs have been *ex vivo* expanded from BM samples collected both at diagnosis and at the different time-points, chosen according to the schedule of BM aspirates of the treatment protocol AIEOP-BFM ALL 2009. For a more meaningful interpretation of data, we also compared the ALL-MSC results with those obtained using MSCs from HDs.

Our data demonstrate that MSCs can both be isolated and successfully expanded from BM of ALL patients not only at disease onset, but also after administration of repeated doses of chemotherapy. These findings suggest that the invasion of the BM by leukemia blasts, especially at disease onset, does not seem to influence the presence of MSCs in the BM microenvironment and does not seem to hamper their *ex vivo* culture when optimal culture conditions are employed. Moreover, the chemotherapy administered to the patients during their early phases of treatment does not seem to affect the frequency of mesenchymal precursors in the BM, since we have been able to isolate ALL-MSCs with 100% efficiency even after more than 2 months of treatment (27 MSCs grown from 27 BM samples of ALL patients harvested at day+78). We also showed that ALL-MSCs, obtained both at diagnosis and subsequent time-points, exhibit similar morphology, immunephenotype and differentiation potential into osteoblasts and adipocytes, as compared with HD-MSCs (Fig. 1 and 4). Altogether, these observations indicate that neither the abundant presence of leukemia cells at diagnosis nor the cytotoxic drugs administered during treatment seem to alter the typical *in vitro* biological properties of BM-derived MSCs, at least according to the characterization assays employed in this study.

ALL-MSCs obtained at any time-point considered displayed a significantly lower proliferative capacity (expressed in terms of PDs), as compared with HD-MSCs (Fig. 2). This is in line with a previous publication [33] showing a defective proliferative capacity of BM-MSCs isolated from 7 children affected by ALL at diagnosis. It has been reported that HD-MSCs isolated from older subjects may display a reduced ability to proliferate and survive *in vitro* as compared with MSCs derived from young healthy donors [36]. Therefore, the reduced proliferative capacity and similar *in vitro* life-span of ALL-MSCs obtained from our pediatric patients as compared with older control subjects (median age of HDs = 21 years) might reflect an intrinsic defect of patient's MSCs. On the other hand, our findings may indicate that the presence of leukemia cells in the BM and the chemotherapy received by the patients may impact on the ability of MSCs to proliferate and expand *in vitro*. When considering ALL-MSCs isolated at diagnosis, our results also suggest that the leukemia itself does not confer to mesenchymal progenitors in the BM any growth advantage or a preferential ability to proliferate, but rather a deficient capacity. This observation does not support the hypothesis that MSCs, a key component of the BM *niche* [11], [12], might in turn be instrumental for efficiently promoting survival and proliferation of leukemia blasts. This interpretation is further confirmed by the reduced ability of ALL-MSCs to support *in vitro* long-term hematopoiesis of healthy subjects, as compared with HD-MSCs. This assay, although cumbersome and limited by a reduced inter-laboratory comparison, has been used previously to assess the competence of MSCs to support *in vitro* hematopoiesis [30]. While not demonstrated *in vivo*, these *in vitro* findings suggest that ALL-MSCs do not display a preferred capacity to sustain the proliferation and expansion of hematopoietic cells. The role played by ALL-MSCs on proliferation of leukemia cells remains unclear, also in view of the technical difficulties in growing ALL cells *in vitro*.

Despite the lower proliferative capacity, ALL-MSCs displayed a life-span *in vitro* similar to that of HD-MSCs and ceased their growth after a number of passages comparable to that of ‘healthy’ cells, by regularly entering into senescence (Fig. 3), as also indicated by cell cycle analysis.

We also demonstrated, to our knowledge for the first time, that ALL-MSCs are equally effective, as HD-MSCs, in inhibiting *in vitro* polyclonally-induced proliferation of allogeneic PBMCs (Fig. 5). Moreover, cytokine and growth factor analysis demonstrated an increase in anti-inflammatory cytokines and a decrease in pro-inflammatory cytokines and growth factors in both HD-MSC and ALL-MSC cultures, this suggesting that MSCs isolated from ALL patients exert similar anti-inflammatory effects as HD-MSCs in this *in vitro* setting.

Our data on the genetic characterization of MSCs show for the first time that MSCs isolated from patients who have been treated with chemotherapy (ALL-MSCs obtained at days +15, +33, +78) do not seem to be more prone to undergo transformation after *ex vivo* culture. Moreover, our ALL-MSCs did not show the presence of leukemia-specific chromosomal translocations (*i.e.* the *TEL/AML* fusion gene in 3 cases and the *MLL/AF4* fusion gene in 2 patients; fig. 6A–B–C). Although experiments were performed in a limited number of patients, it might be speculated that the first oncogenic hit responsible for leukemia development might occur downstream a common precursor between HSCs and mesenchymal progenitors and, consequently, MSCs would not belong to or derive from the original malignant clone [37]–[39]. The possibility that human MSCs may derive from the malignant clone in hematological malignancies is a debated issue that has been investigated in several papers with conflicting results [5]–[7], [22]–[24], [38]–[41]. A possible pathogenetic role of BM-MSCs in lymphoproliferative disorders has been suggested by some authors, in particular for what concerns survival, drug resistance, and proliferation of pathologic lymphocytes [21]–[24], [41]. Also the presence of chromosomal abnormalities in MSCs from hematological malignances has been tested by several authors. In particular, the question of whether BM-MSCs from childhood leukemia harbor and express leukemia-specific fusion genes has been addressed in 2 studies. Shalapour *et al.* reported the same chromosomal translocations (*TEL-AML1*, *E2A-PBX1*, *MLL* rearrangement) that had been detected in leukemia cells in BM-derived MSCs (in a proportion varying between 10% and 54%) in 10 out of 10 ALL patients analyzed [23]. Menendez *et al.* documented that BM-derived MSCs from infants with *MLL-AF4*+ acute leukemia harbor and express the *MLL-AF4* fusion gene, while they did not find fusion genes in BM-MSCs of childhood leukemias carrying *TEL-AML1*, *BCR-ABL*, *AML1-ETO*, *MLL-AF9*, *MLL-AF10*, *MLL-ENL* translocations or hyperdiploidy. The authors interpreted their results claiming that *MLL-AF4* itself is not sufficient for MSC transformation and the expression of *MLL-AF4* in MSCs is compatible with a mesenchymal phenotype, suggesting a differential impact in the hematopoietic system and mesenchyme [21]. More recently, Campioni *et al.* found normal karyotype in BM-MSCs isolated from ALL and chronic lymphocytic leukemia patients and underlined the importance of excluding the persistence of contaminating hematopoietic cells in mesenchymal cultures, which can lead to a misinterpretation of data on the presence of cytogenetic aberrations in *ex vivo* expanded MSCs [37]. In line with this latter study, our findings suggest that BM-MSCs isolated from ALL pediatric patients are not tumor-related and do not derive from the original malignant clone, although our analysis has the relevant limitation of the small number of analyzed cases carrying specific chromosomal alterations.

Moreover, we show that the chemotherapy treatment administered to the children do not seem to favor the development of malignant transformation in BM-MSCs at any time-point considered.

Correlations between clinical groups of patients (for example high-risk vs. non high-risk patients, patients in remission vs. relapsed) and the characteristics of their *ex vivo* expanded MSCs would potentially unravel interesting insights on leukemia biology and its surrounding BM environment. However, this analysis is limited by the relatively small number of patients enrolled in the study which does not allow for a meaningful statistical comparison between different clinical groups.

In conclusion, this study provides a comprehensive characterization of BM-derived MSCs obtained from ALL pediatric patients not only at diagnosis, but also during treatment. Our results indicate that ALL-MSCs maintain all the typical mesenchymal properties, except for a reduced ability to proliferate and to support long-term hematopoiesis *in vitro*. Moreover, the chemotherapy administered to the patients *in vivo* does not seem to interfere with MSC functional and biological characteristics. The absence of genetic abnormalities in ALL-MSCs argues for an origin of mesenchymal progenitors different from that of the malignant clone.
